# ON THE USE OF NONPARAMETRIC BOUNDS FOR CAUSAL EFFECTS IN NULL RANDOMIZED TRIALS

**DOI:** 10.1093/aje/kwab153

**Published:** 2021-05-21

**Authors:** Erin E Gabriel, Michael C Sachs

**Affiliations:** Department of Medical Epidemiology and Biostatistics, Karolinska Institutet, Stockholm, Sweden

The goal of randomized experiments is to estimate the causal effect of an intervention on a clinically relevant outcome. When study subjects are missing outcome information due to factors related to the intervention, compliance, or the outcome, the causal effect is not identifiable from the observed data alone (1). When there is no missing data, randomization allows identification of the effect of assignment to the intervention, sometimes called the intent-to-treat effect; this is only equivalent to the intervention effect if subjects comply with their assigned intervention as directed. When this is not the case, the intervention effect is nonidentifiable, even with no missing data, without making additional assumptions (2).

The Danish randomized trial studying the effect of mask recommendation (DANMASK-19) was widely discussed in the media (3). In the study, 3,030 participants were randomly assigned to the mask group, and 2,994 to the control group; 638 and 524, respectively, did not complete the study. The reported infection risk difference comparing the mask recommendation arm with the control arm was −0.3% (95% confidence interval: −1.2, 0.4). The primary analysis excluded subjects missing data (a complete-case analysis), and the authors used multiple imputation with additional covariates in secondary analyses. The complete-case analysis is valid if the missingness is completely random, and the imputation analysis is valid if the missingness depends only on included observed covariates. However, neither produce valid estimates of the causal effect of the mask recommendation when the missingness depends on unmeasured factors, which is always a possibility.

We aimed to analyze this trial from a causal inference perspective. Our interest is in the causal risk difference (i.e., the difference in the probability of infection given all subjects had received the intervention minus the probability had they not). Neither the causal effect of mask recommendation nor the effect of mask wearing is estimable in this study without unverifiable assumptions. We instead derived upper and lower bounds for these 2 effects of interest. We aimed to estimate the causal effect of the recommendation to wear masks for those that wear them, not the effect on transmission to others, which is thought to be larger.

The causal model is illustrated by the direct acyclic graph (DAG), presented in Figure 1A; arrows directed from one variable to another indicate a causal relationship. Participants are randomly assigned to receive or not the recommendation to wear a mask, so there are no arrows to that node. Whether or not a subject wears a mask is affected not only by the recommendation but also potentially by unmeasured factors. Infection is affected by mask use (but not directly by the recommendation) and potentially unmeasured factors. Being observed at the end of follow-up is affected by all variables, including unmeasured factors and the unblinded recommendation. The complete-case analysis conditions on being observed, opening a path from the exposures to the outcome, which may induce the observed risk differences in the complete-case analysis.

**Figure 1:**
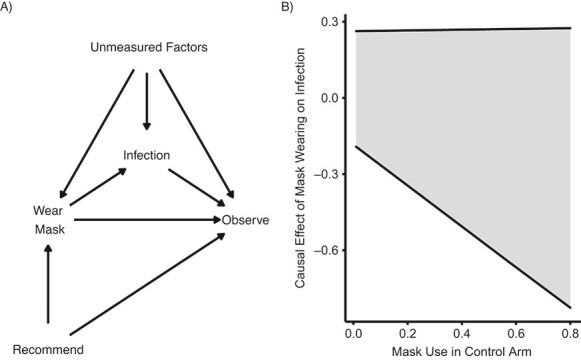
A) Directed acyclic graph for the DANMASK-19 study (3). B) The shaded area between the solid lines represents the bounds on the causal risk difference as a function of the unknown proportion of mask usage in the control arm.

A suggested practice is to assume that all missing participants in the recommendation arm were infected and none in the control arm were, called worst-case imputation (4). This paired with the opposite, best-case imputation, gives bounds for the risk difference for recommendation of −18% to 21%. Based on these bounds, we cannot definitively rule out null or small to moderate effects of mask recommendation in the direction of benefit or harm. A recent publication describes methods for bounding treatment effects in settings similar to the DANMASK-19 study, both for the effect of mask recommendation and of mask wearing (5). It can be shown that the best-case/worst-case bounds are the tightest nonparametric bounds for the causal effect of recommendation under this direct acyclic graph, without making further assumptions. The bounds and details of our calculations are available in Web Appendix 1 (available at https://doi.org/10.1093/aje/kwab153).

To bound the causal effect of mask wearing, we do not have all the information we need from the published text because the investigators did not ascertain mask use in the control arm. Thus, we must impute the infection rates in the control arm conditional on being observed and wearing or not wearing masks, and the proportion wearing masks among those assigned to the control arm who completed the trial. We allow these quantities to vary and construct the bounds, shown in Figure 1B. The bounds and details of these calculations are available in Web Appendix 2.

The bounds are wide, and the variable determining the probability of wearing a mask has a more important effect on the width of the bounds compared with the other variables (not shown in figure). Under any of the assumed proportions, this study provides little information regarding the causal effect of mask wearing on the risk of infection. Even moderate positive effects cannot be ruled out. This is in contrast to the conclusions that the recommendation to wear masks did not reduce incidence of serious acute respiratory syndrome coronavirus 2 (SARS-CoV-2) infection in mask wearers, based on the lack of a statistically significant effect, or that the data are compatible with effects that cannot be very large, based on confidence intervals.

To ensure validity of causal effect estimates in a randomized experiment, every effort should be made to avoid missing data (6). When missing data are unavoidable, bounds can be used to quantify the uncertainty in the causal effect of an interest, in addition to complete-case analysis and using assumption-driven sensitivity analysis. Nonparametric bounds can often be computed using reported summary statistics, as we have shown here. Worst-case estimates are common in randomized clinical trials with missing data that find significant effects, but bounds should be used even when null results are found to put findings in perspective. Confidence intervals account for uncertainty due to sampling variability, but uncertainty due to unmeasured confounding introduced by nonignorable missingness or noncompliance are relatively greater threats to causal inference. When confidence intervals alone are used, particularly in the reporting of results from null and controversial trials, misunderstandings are bound to occur.

## Supplementary Material

Web_Material_kwab153
