# Supplementary material for: ON THE USE OF NONPARAMETRIC BOUNDS FOR CAUSAL EFFECTS IN NULL RANDOMIZED TRIALS
Source: Am J Epidemiol. 2021 May 21;190(10):2231. doi: 10.1093/aje/kwab153 (PMC8485145; doi:10.1093/aje/kwab153)
Supplement: Web_Material_kwab153 [file web_material_kwab153.pdf]

Web Material

On the Use of Nonparametric Bounds for Causal Effects in Null Randomized Trials

Erin E Gabriel and Michael C Sachs

Web Appendixes 1 and 2

Web Appendix 1

In general, the effect of recommendation is bounded between the lower bound = p{Y=0,O=1|R=0} + p{Y=1,O=1|R=1} – 1 and the upper bound = 1 – p{Y=0,O=1|R=1} – p{Y=1,O=1|R=0}, where Y=1 represents infection, O=1 means observed, O=0 means missing, and R=0/1 represents assigned to the control/mask recommended group. It can be shown that the best-case/worst-case bounds as described above are the tightest nonparametric bounds for the causal effect of recommendation under the model in Figure A of the main text, without making further assumptions.

In the R code below, the probabilities are pio_r = pr(infection = i, observed = o | recommend = r). The code below contains two different expressions for the best-case worst-case bounds, and they are equivalent.

```
rho.bounds <- function (p01_0 = NULL,
                        p01_1 = NULL,
                        p11_0 = NULL,
                        p11_1 = NULL) {
  data.frame(lower = pmax(p01_0 + p11_1 - 1),
             upper = pmin(-p01_1 - p11_0 + 1))
}


best.worst.rho <- function() {
  c(
    lower = (42 + 0 * 638) / 3030 - (53 + 1 * 524) / 2994,
    upper = (42 + 1 * 638) / 3030 - (53 + 0 * 524) / 2994
  )
}


rho.bounds(
  p01_0 = ((2994 - 524) / 2994) * ((2994 - 524 - 53) / (2994 - 524)),
  p11_0 = ((2994 - 524) / 2994) * (53 / (2994 - 524)),
  p01_1 = ((3030 - 638 - 42) / (3030 - 638)) * ((3030 - 638) / 3030),
  p11_1 = (42 / (3030 - 638)) * ((3030 - 638) / 3030)
)

##        lower     upper
## 1 -0.1788574 0.2067204

best.worst.rho()

##      lower      upper
## -0.1788574  0.2067204
```

Web Appendix 2

To bound the causal effect of mask wearing, let pxy.r=p{X=x, Y=y, O=1|R=r}, where y,x,r can be 0 or 1, Y, O, R are the random variables defined as above, and X=1 represents mask use, and X=0 no mask use. We show in (1) that the effect of mask wearing is bounded between the lower bound =

max(p00.1 + p11.1 – 1,
p00.0 + p11.1 – 1,
p00.1 + p11.0 – 1,
p00.0 + p11.0 – 1,
2×p00.1 + p01.0 + p11.0 + p11.1 – 2,
2×p00.0 + p01.1 + p11.0 + p11.1 – 2,
p00.0 + p00.1 + p10.0 + 2×p11.1 – 2,
p00.0 + p00.1 + p10.1 + 2×p11.0 – 2)

and the upper bound =

min(- p10.0 - p01.0 + 1,
- p10.0 - p01.1 + 1,
- p10.1 - p01.0 + 1,
- p10.1 - p01.1 + 1,
- p00.0 - p10.0 - p10.1 - 2×p01.1 + 2,
- p00.1 - p10.0 - p10.1 - 2×p01.0 + 2,
- 2×p10.0 - p01.0 - p01.1 - p11.1 + 2,
- 2×p10.1 - p01.0 - p01.1 - p11.0 + 2).

In the code below, the probabilities are pmio_r = pr(mask = m, infect = i, observed = o | recommend = r)

```
mu.bounds <-
  function (p001_0 = NULL,
            p001_1 = NULL,
            p101_0 = NULL,
            p101_1 = NULL,
            p011_0 = NULL,
            p011_1 = NULL,
            p111_0 = NULL,
            p111_1 = NULL) {
    data.frame(
      lower = pmax(
        p001_1 + p111_1 - 1,
        p001_0 + p111_1 - 1,
        p001_1 + p111_0 - 1,
        p001_0 + p111_0 - 1,
        2 * p001_1 + p011_0 + p111_0 + p111_1 - 2,
        2 * p001_0 + p011_1 + p111_0 + p111_1 - 2,
        p001_0 + p001_1 + p101_0 + 2 * p111_1 - 2,
        p001_0 + p001_1 + p101_1 + 2 * p111_0 - 2
      ),
      upper = pmin(
        -p101_0 - p011_0 + 1,
```

```
        -p101_1 - p011_0 + 1,
        -p101_0 - p011_1 + 1,
        -p101_1 - p011_1 + 1,
        -p001_0 - p101_0 - p101_1 - 2 * p011_1 + 2,
        -p001_1 - p101_0 - p101_1 - 2 * p011_0 + 2,
        -2 * p101_0 - p011_0 - p011_1 - p111_1 + 2,
        -2 * p101_1 - p011_0 - p011_1 - p111_0 + 2
      )
    )
  }
```

Since the investigators of the mask trial (2) did not report mask use in the control arm, we must make some assumptions to compute these bounds. Assumption 1: p(I = i | M = 1-m, O = 1, R = 0) = same for R = 1. Then we assume different values for P(mask | control, obs), P(infection | no mask, control, obs), P(infection | mask, control, obs) and compute the bounds for each assumed value.

```
beefig <- function(pm.o, pa1, pa0) {
  #
  # im
  pa1.11 <- pa1 # 0.018
  pa1.10 <- pa0 # 0.012
  pa1.00 <- (1 - pa0)
  pa1.01 <- (1 - pa1)


  # pmio_r
  bpmu <- mu.bounds(
    p001_0 = 0.82 * ((1 - 0.021) - pm.o * pa1.01),
    p001_1 = 0.054,
    p011_0 = 0.82 * ((0.021) - pm.o * pa1.11),
    p011_1 = 0.001,
    p101_0 = 0.82 * ((1 - 0.021) - (1 - pm.o) * pa1.00),
    p101_1 = 0.72,
    p111_0 = 0.82 * ((0.021) - (1 - pm.o) * pa1.10),
    p111_1 = 0.013
  )

  c(bpmu$lower, bpmu$upper)

}


pmu <- seq(0.01, 0.83, by = .01)
pa1 <- seq(0.0, .2, by = .01)
pa0 <- seq(0.0, .2, by = .01)

mubounds1 <-
  data.frame(rbind(cbind(1, pmu, t(
```

```
    sapply(pmu, beefig, .018, .012)
  )),
  cbind(2, pa1, t(
    sapply(pa1, function(x)
      beefig(.15, x, .012))
  )),
  cbind(3, pa0, t(
    sapply(pa0, function(x)
      beefig(.15, .018, x))
  ))))

colnames(mubounds1) <- c("group", "variable", "lower", "upper")
mubounds1$group <-
  factor(
    mubounds1$group,
    levels = 1:3,
    labels = c("P(mask | control, obs)", "P(infection | no mask, control,
obs)",
               "P(infection | mask, control, obs)")
  )
```

References

1. Gabriel EE, Sjölander A, Sachs MC. Nonparametric bounds for causal effects in imperfect randomized experiments [preprint]. *arXiv preprint*s. 2020. (https://arxiv.org/abs/2010.05220). Accessed May 13, 2021.

2. Henning Bundgaard H, Bundgaard JS, Raaschou-Pedersen DET, et al. Effectiveness of Adding a Mask Recommendation to Other Public Health Measures to Prevent SARS-CoV-2 Infection in Danish Mask Wearers – A Randomized Controlled Trial. **Ann Intern Med**. 2020 Nov 18: M20-6817. doi:10.7326/M20-6817.
